# Evidence for Transient, Uncoupled Power and Functional Connectivity Dynamics

**DOI:** 10.1002/hbm.70179

**Published:** 2025-03-04

**Authors:** Rukuang Huang, Chetan Gohil, Mark Woolrich

**Affiliations:** ^1^ Oxford Centre for Human Brain Activity (OHBA), Wellcome Centre for Integrative Neuroimaging, Department of Psychiatry University of Oxford UK

**Keywords:** dynamic functional connectivity, machine learning, magnetoencephalography, resting‐state networks, task evoked response

## Abstract

There is growing interest in studying the temporal structure in brain network activity, in particular, dynamic functional connectivity (FC), which has been linked in several studies with cognition, demographics and disease states. The sliding window approach is one of the most common approaches to compute dynamic FC. However, it cannot detect cognitively relevant and transient temporal changes at time scales of fast cognition, that is, on the order of 100 ms, which can be identified with model‐based methods such as the HMM (Hidden Markov Model) and DyNeMo (Dynamic Network Modes) using electrophysiology. These new methods provide time‐varying estimates of the ‘power’ (i.e., variance) and of the functional connectivity of the brain activity, under the assumption that they share the same dynamics. But there is no principled basis for this assumption. Using a new method that allows for the possibility that power and FC networks have different dynamics (Multi‐dynamic DyNeMo) on resting‐state magnetoencephalography (MEG) data, we show that the dynamics of the power and the FC networks are not coupled. Using a (visual) task MEG dataset, we show that the power and FC network dynamics are modulated by the task, such that the coupling in their dynamics changes significantly during the task. This work reveals novel insights into evoked network responses and ongoing activity that previous methods fail to capture, challenging the assumption that power and FC share the same dynamics.

## Graphical abstract

Amplitude envelope data were extracted from source‐reconstructed MEG data. A novel generative model, M‐DyNeMo, was used to extract uncoupled power and FC network dynamics, which responded differently to task stimuli.

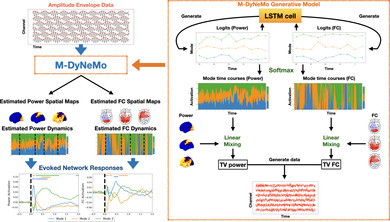

**Summary**

- Power and FC networks with similar spatial patterns show independent dynamics at rest. We show that recognisable resting‐state networks with independent power and FC dynamics can be reproducibly inferred across different subjects, datasets and parcellations.
- The evoked dynamics of power and FC are not the same. In a visual perception task, we observe that the evoked response in FC (i.e., time course of FC response) differs from that of power. This is reproduced over two independent visual perception task datasets.
- A task structure induces a significant change in coupling between power and FC. We show evidence that when there is a task structure in the recording, this modulates the power and FC dynamics, which induce a significant change in coupling.

## 1 Introduction

Recently, researchers have adopted the view that cognitive tasks are performed not by individual brain regions working in isolation, but by networks of several brain areas that communicate with each other. A metric often used to characterise this communication is functional connectivity (FC), which is defined as the temporal correlation of functional activity between spatially remote regions (Friston 1994). The study of FC has become increasingly popular as evidence suggests that FC is related to underlying neural activity (Nir et al. 2008) and significant differences have been observed in FC between healthy and diseased states (Greicius 2008; Heine et al. 2012; Menon 2011).

Traditionally, it was typically assumed that the strength of interactions between regions is constant over time (stationary) during resting‐state scans. However, given the brain's dynamic nature (Rabinovich, Friston, and Varona 2012), an increasingly important perspective is how functional networks change over time. One of the most common approaches to compute dynamic FC is the sliding window approach (Allen et al. 2014; Chang and Glover 2010; Liuzzi et al. 2019). Clustering methods like K‐means clustering are followed to identify reproducible and transient patterns of FC states (Allen et al. 2014).

Despite its popularity, the sliding window approach is a heuristic approach in which the choice of hyperparameters can have an impact on the interpretation of results. More importantly, sliding window methods are deficient at adapting to fast dynamics in the data and finding sub‐second transitions in brain dynamics, which have been shown to be present in electrophysiological data like magnetoencephalography (MEG) (Vidaurre et al. 2016, 2018). This is due to a fixed window size being assumed, with larger windows being insensitive to fast changes and smaller windows giving noisier estimates of FC.

Recent advances in the field of machine learning provide methods for inferring dynamic FCs in a data‐driven way. An example is the recently proposed Dynamic Network Modes model (DyNeMo, Gohil et al. 2022) which allows for coactivation of multiple networks simultaneously. It has been shown that DyNeMo can infer dynamic FC more accurately than state‐based models, such as the Hidden Markov Model (Gohil et al. 2022), and can identify more parsimonious dynamic network descriptions when studying task data (Gohil et al. 2024b).

One important assumption that underlies DyNeMo is that time‐varying (TV) variance (which we will refer to equivalently as ‘power’) and FC follow the same temporal dynamics, but there is no principled reason to assume that fluctuation in connections between brain regions should be coupled with that in power of individual brain regions. This motivates us to present Multi‐dynamic Network Modes (M‐DyNeMo), an extension to DyNeMo, to infer potentially uncoupled power and FC dynamics.

In this paper, with resting‐state MEG data, we present evidence of separate power and FC dynamics given by the traditional sliding window approach, and show that plausible networks of separate dynamics can be inferred by M‐DyNeMo. We further demonstrate that the networks are reproducible across different subjects, datasets and parcellations, validating the robustness of M‐DyNeMo on MEG data. Using visual task data, M‐DyNeMo reveals distinct evoked network responses of power and FC dynamics to visual stimuli. We also show evidence for task‐induced coupling between power and FC dynamics.

## 2 Materials and Methods

### 2.1 Dataset

Two real MEG datasets are used in this paper. The first dataset is part of the UK MEG Partnership acquired using a 275‐channel CTF MEG scanner at a sampling frequency of 1.2 kHz. This dataset includes (eyes open) resting‐state recordings of 65 subjects and visual task recordings of 67 subjects, of which 63 subjects have both resting‐state and task MEG recordings. We refer to this dataset as the **MEGUK** dataset.

The second dataset is a visual task MEG dataset collected using an Elekta Neuromag Vectorview 306 scanner at a sampling frequency of 1 kHz. In this dataset, 19 participants (11 males, 8 females, aged 23–37 years) were scanned six times, during which they were presented with images of famous, unfamiliar or scrambled faces. Each recording session is around 7.5 min and contains approximately 200 trials which are evenly split across three different images. During the experiment, participants were also asked to press one of two keys based on how symmetric they regarded each image. This is to ensure the participants focus on the images. The MaxFiltered data are publicly available, and we direct the readers to the original paper (Wakeman and Henson 2015) for more details on the experimental design and data collection. We refer to this dataset as the **Wakeman–Henson** dataset.

#### 2.1.1 Data Preprocessing

Both datasets are preprocessed using the *osl‐ephys* package (van Es et al. 2024) with the same pipeline. A band‐pass filter from 0.5 Hz to 125 Hz and a notch filter with 50 Hz and 100 Hz were applied to the raw data. Then, the data were downsampled to 250 Hz, after which automatic bad segment and bad channel detection were used to remove abnormally noisy segments and channels of the recordings. A final independent component analysis (ICA) step with 64 components was used to remove noise.

#### 2.1.2 Source Reconstruction

Coregistration and source reconstruction were done using osl‐ephys. MEG data were first coregistered with the structural MRI data and digitised headshape points acquired with a Polhemus pen of each subject. Then, the data were source reconstructed onto an 8‐mm isotropic grid using a linearly constrained minimum variance (LCMV) beamformer (Van Veen and Buckley 1988; van Veen et al. 1997).

#### 2.1.3 Parcellation

Two atlases were used for parcellating the MEGUK dataset; one is the Giles parcellation with 38 regions of interest (ROIs) and the other is the Glasser parcellation with 52 ROIs, referred to as **MEGUK‐38** (in which only the resting‐state recordings were used in this paper) and **MEGUK‐52** respectively. The Wakeman–Henson dataset was parcellated with the Giles parcellation to 38 ROIs. Finally, the symmetric multivariate leakage reduction (Colclough et al. 2015) and sign‐flipping algorithm (Vidaurre et al. 2016) were applied.

#### 2.1.4 Preparation Before Model Training

Extra steps were taken to help the convergence of model training. Data were band‐pass filtered between 1 and 45 Hz to concentrate the data in the frequency range where neural activity is the most prominent before the amplitude envelope signals were extracted using the Hilbert transform (Feldman 2011). A moving average of window size of 25 samples (100 ms at 250 Hz) and step size of one sample (4 ms at 250 Hz) was applied to smooth the amplitude envelope data. Then, a standardisation step (z‐transform) was applied to ensure zero mean and unit variance for each channel. This was done independently for each recording session. Notice this normalisation step will remove the heterogeneity in (static) power information between recording sessions, but the temporal structure of power fluctuation, which is the object of interest in this paper, remains unchanged within each session. Finally, we orthogonalised the data with a full‐rank PCA (See Section null. for details).

### 2.2 Measure of Power and FC

In this paper, variance, instead of mean activation, of the amplitude of the recording signals is used as a measure of power. This is because we adopt the so‐called *zero‐mean model* (in Section 2.4), which has been widely used in previous literature (Gohil et al. 2022; Vidaurre et al. 2016, 2018). Correlation between amplitude signals is used as a measure of FC.

### 2.3 Sliding Window Approach

The aim of this method is to use a model‐free approach to partition the time dimension into a finite number of states according to dynamic changes in either power or FC. The heuristic approach is a combination of sliding window and K‐means clustering. The steps include:

- Apply sliding window with a window size of 500 samples (2 s at 250 Hz) and a step size of 10 samples (40 ms at 250 Hz).
- For each window, calculate the standard deviations of the envelope time courses for all channels/parcels and calculate the correlations between the envelope time courses between channels/parcels.
- Apply K‐means clustering algorithm to the time‐varying correlations and standard deviations separately.

Each time point is assigned to a cluster, which in our case can be thought of as a state, according to either TV power or FC. Hence, we have formed two state time courses for power and FC separately.

### 2.4 Generative Model of M‐DyNeMo

**Notations**

- $\left[n\right]=\left\{1,\ldots n\right\}$ for n∈N.
- x is a column vector.
- A is a matrix.
- $I_{n}$ is an n×n identity matrix.
- $x_{1:n}=\left\{x_{i}:i\in \left[n\right]\right\}$.

In this section, we formulate the generative model of M‐DyNeMo. Conceptually, M‐DyNeMo learns two basis sets of spatial configurations of networks (called *modes*), one for power (where the network is a spatial map over brain regions) and another for the FC (where the network is an edge‐wise connectome). The underlying assumption is that the TV power/FC is a time‐varying linear mixture of the basis set. The time series of mixing coefficients characterises the dynamics of the modes and is referred to as the *mode time courses*. Fluctuations in power and FC can be described independently via dynamics in the mixing of their respective basis sets.

Similar to DyNeMo, it is assumed that the data is generated by a multivariate Gaussian distribution with zero mean. Let $x_{1:T}$ be the observed data, where T is the number of samples/timestamps, $x_{t}$ is a vector of length and $N_{c}$ is the number of channels. Then,

(1) $$x_{t}∼N\left(0,C_{t}\right)\;\forall t\in \left[T\right]$$

independently, where $C_{t}$ is the time‐varying covariance matrix of the data at time t.

In M‐DyNeMo, it is further assumed that $C_{t}$ can be decomposed into

(2) $$C_{t}=G_{t}F_{t}G_{t},$$

where $G_{t}$ is a diagonal matrix with strictly positive diagonal entries and $F_{t}$ is a positive‐definite matrix with ones on the diagonal. In particular, the diagonal elements of $G_{t}$ are the time‐varying standard deviations and the off diagonal elements of $F_{t}$ are the time‐varying correlations of the data at time t. The time‐varying standard deviations and correlations are assumed to be generated by a linear mixture of a finite number of modes:

(3) $$\begin{array}{c} G_{t}=\sum_{j=1}^{J^{1}}\alpha_{\mathrm{jt}}E_{j},\\ F_{t}=\sum_{j=1}^{J^{2}}\beta_{\mathrm{jt}}R_{j}. \end{array}$$

Here, $E_{j}$ is a diagonal matrix with positive diagonal entries, and $R_{j}$ is a positive‐definite matrix with ones on the diagonal. Notice here that the number of modes $J^{1},J^{2}$ can be different. The mixing coefficients $\alpha_{\mathrm{jt}}$ and $\beta_{\mathrm{jt}}$ are generated through a softmax transformation of latent probabilistic variables $\theta_{1:T}^{1},\theta_{1:T}^{2}$, respectively, whose prior distribution is parameterised by an RNN:

(4) $$\begin{array}{c} p\left(\theta_{1:T}^{1,2}\right)=\prod_{t=2}^{T}p\left(\theta_{t}^{1,2}|\theta_{1:t-1}^{1,2}\right)\\ =\prod_{t=2}^{T}\prod_{k=1}^{2}p\left(\theta_{t}^{k}|\theta_{1:t-1}^{1,2}\right)\\ =\prod_{t=2}^{T}\prod_{k=1}^{2}N\left(\theta_{t}^{k}|\mu_{\theta_{t}^{k}}\left(\theta_{1:t-1}^{1,2}\right),\sigma_{\theta_{t}^{k}}^{2}\left(\theta_{1:t-1}^{1,2}\right)I_{J^{k}}\right), \end{array}$$

where
(5)

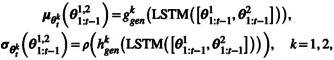

and $\left[\theta_{1:t-1}^{1},\theta_{1:t-1}^{2}\right]$ represents concatenating $\theta_{1:t-1}^{1},\theta_{1:t-1}^{2}$ along the mode dimension. Here, LSTM is a long short‐term memory recurrent neural network (Hochreiter and Schmidhuber 1997), $g_{\mathrm{gen}}^{k},h_{\mathrm{gen}}^{k}$ are learnable linear transformations (dense layers) and ρ is the softplus activation.

**FIGURE A.1:**
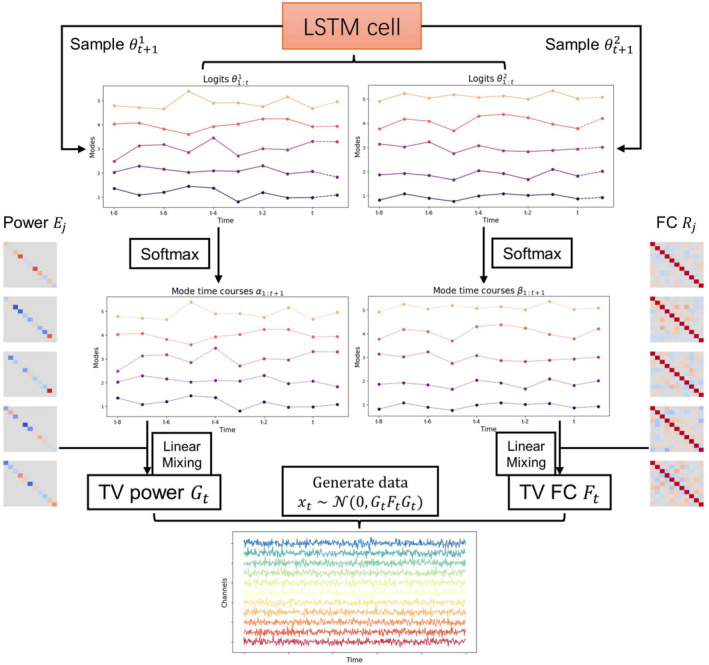
Generative model of M‐DyNeMo. Historic logits $\theta_{1:t}^{1}$ and $\theta_{1:t}^{2}$ are concatenated and passed to the LSTM cell. The outputs of the LSTM cell are the parameters of the distribution from which we sample the future logits $\theta_{t+1}^{1}$ and $\theta_{t+1}^{2}$. The sampled logits are concatenated to the original logit sequences and the process is repeated. The generated sequences of logits are normalised with a softmax transformation and we get the mode time courses $\alpha_{1:T},\beta_{1:T}$. Diagonal matrices $E_{j}$ and correlation matrices $R_{j}$ are linearly mixed with $\alpha_{1:T}$ and $\beta_{1:T}$ respectively to give the time‐varying power $G_{t}$ and FC $F_{t}$. Finally the observed data are generated through the multivariate Gaussian distribution with time‐varying covariance $G_{t}F_{t}G_{t}$.

With this generative model, each of the latent logits $\theta_{t}^{1},\theta_{t}^{2}$ is generated by the history of both logits. This allows information to be communicated between the two different time courses in the generative model. An overview of the generative model is illustrated in Appendix Figure A.1.

#### 2.4.1 Orthogonalisation With PCA

Due to model complexity, the stability of training the generative model above is suboptimal, especially on small datasets. We found in practice that orthogonalising the data, that is, removing the correlation between channels, improved the convergence of model training significantly. To this end, a full‐rank principal component analysis (PCA) was applied to the data. Notice that the PCA‐projected channels are linear combinations of the original channels, which makes separating the dynamics of power and FC in this projected space nontrivial. Hence, we instead modify the generative model and incorporate the PCA projection matrix into the generative model specified above. This allows easy interpretation of the learnt observation model parameters.

Formally, let $W\in R^{N_{c}\times N_{c}}$ be the matrix with eigenvectors of the covariance matrix of the data on the rows, then the PCA‐transformed data ${\tilde{x}}_{t}=Wx_{t}$ at time t has the following distribution, under the M‐DyNeMo generative model:

(6) $${\tilde{x}}_{t}∼N\left(0,{\tilde{C}}_{t}\right),$$

where ${\tilde{C}}_{t}=WC_{t}W^{T}$. Due to the fact that W is full‐rank and hence $W^{T}W=I_{N_{c}}$,

(7) $$\begin{array}{c} {\tilde{C}}_{t}=WC_{t}W^{T}\\ =WG_{t}F_{t}G_{t}W^{T}\\ =\left(WG_{t}W^{T}\right)\left(WF_{t}W^{T}\right)\left(WG_{t}W^{T}\right)\\ ≔{\tilde{G}}_{t}{\tilde{F}}_{t}{\tilde{G}}_{t}. \end{array}$$

Then, the projection matrix W can be passed in into linear combination equations:

(8) $$\begin{array}{c} {\tilde{G}}_{t}=WG_{t}W^{T}=\sum_{j=1}^{J^{1}}\alpha_{\mathrm{jt}}{\text{WE}}_{j}W^{T},\\ {\tilde{F}}_{t}=WF_{t}W^{T}=\sum_{j=1}^{J^{2}}\beta_{\mathrm{jt}}WR_{j}W^{T}. \end{array}$$

Now we have defined the generative model in the PCA‐transformed space with the observation model parameters $\theta_{\mathrm{obs}}$ in the original space.

### 2.5 Training M‐DyNeMo

In this work, data from different recording sessions were concatenated over the time dimension and the model was trained on the concatenated data, that is, the inferred networks are shared across recording sessions and the model gives a group‐level description of the data. It is possible to get individual‐level networks with a method called dual estimation, which involves freezing the temporal model (model time courses) of the group‐level model and training the model on individual data (Gohil et al. 2024a). The goal of training is to infer the posterior distribution of the latent variables $\theta_{1:T}^{1},\theta_{1:T}^{2}$ as well as the observation model parameters $\theta_{\mathrm{obs}}=\left\{E_{j}:j\in \left[J^{1}\right]\right\}\cup \left\{R_{j}:j\in \left[J^{2}\right]\right\}$. This is achieved by minimising the variational free energy (Blei, Kucukelbir, and McAuliffe 2017).

In practice, it is infeasible to feed the whole time series to the LSTM due to memory restrictions. More importantly, although the use of LSTM mitigates the issue of gradient explosion and vanishing to some extent, it is still an issue when the sequence is long. Hence, we separate the data into $N_{\mathrm{seq}}$ sequences of length $T_{\mathrm{seq}}=200$ samples (so that $\left\lfloor T/T_{\mathrm{seq}}\right\rfloor =N_{\mathrm{seq}}$). For M‐DyNeMo, the loss for each sequence n is

(9) $${ℒ}_{n}=-\underset{\mathrm{LL}}{\underbrace{\sum_{t=1}^{T_{\mathrm{seq}}}E_{q\left(\theta_{t}^{1,2}\right)}\left[\log p\left({\tilde{x}}_{t},\theta_{t}^{1,2}|\theta_{\mathrm{obs}}\right)\right]}}+\underset{\mathrm{KL}}{\underbrace{\sum_{t=2}^{T_{\mathrm{seq}}}\sum_{k=1}^{2}D_{\mathrm{KL}}\left(q\left(\theta_{t}^{k}\right)\|p\left(\theta_{t}^{k}|\theta_{1:t-1}^{1,2}\right)\right)}},$$

where $D_{\mathrm{KL}}\left(\cdot \|\cdot \right)$ is the Kullback–Leibler divergence (Kullback and Leibler 1951) and we have used the mean field approximation:

(10) $$\begin{array}{c} q\left(\theta_{1:T_{\mathrm{seq}}}^{1,2}\right)=\prod_{t=1}^{T_{\mathrm{seq}}}\prod_{k=1}^{2}q\left(\theta_{t}^{k}\right),\\ =\prod_{t=1}^{T_{\mathrm{seq}}}\prod_{k=1}^{2}N\left(\theta_{t}^{k},m_{\theta_{t}^{k}}|s_{\theta_{t}^{k}}^{2}I_{J^{k}}\right),\\ m_{\theta_{t}^{k}}=g_{\inf}^{k}\left(\text{BiLSTM}\left({\tilde{x}}_{1:T_{\mathrm{seq}}}\right)\right),\\ s_{\theta_{t}^{k}}=\rho \left(h_{\inf}^{k}\left(\text{BiLSTM}\left({\tilde{x}}_{1:T_{\mathrm{seq}}}\right)\right)\right), \end{array}$$

The learning of variational parameters were amortised by learning a map from the data to the variational parameters $m_{\theta_{t}^{k}},s_{\theta_{t}^{k}}$ using bi‐directional LSTM BiLSTM and learnable linear transformations $g_{\inf}^{k},h_{\inf}^{k}$. This allows not only scalable training, but also direct inference on unseen data. During training, mini‐batches of sequences were shuffled and at each step of optimisation, each mini‐batch of sequences was used to calculate the loss and gradient. Gradient descent based algorithm (in this paper ADAM, Kingma and Ba 2014), together with the reparameterisation trick (Kingma and Welling 2013) to pass gradient through the LL term, were used to minimise the loss.

Several tricks were used to help with the convergence of training and minimisation of the loss. See Appendix Section B for more details.

## 3 Results

In this section, we study three MEG datasets (Section 2.1): MEGUK‐38 (resting‐state), MEGUK‐52 and Wakeman–Henson.

### 3.1 Sliding Window Approach Reveals Distinct Power and FC Dynamics

**FIGURE 1:**
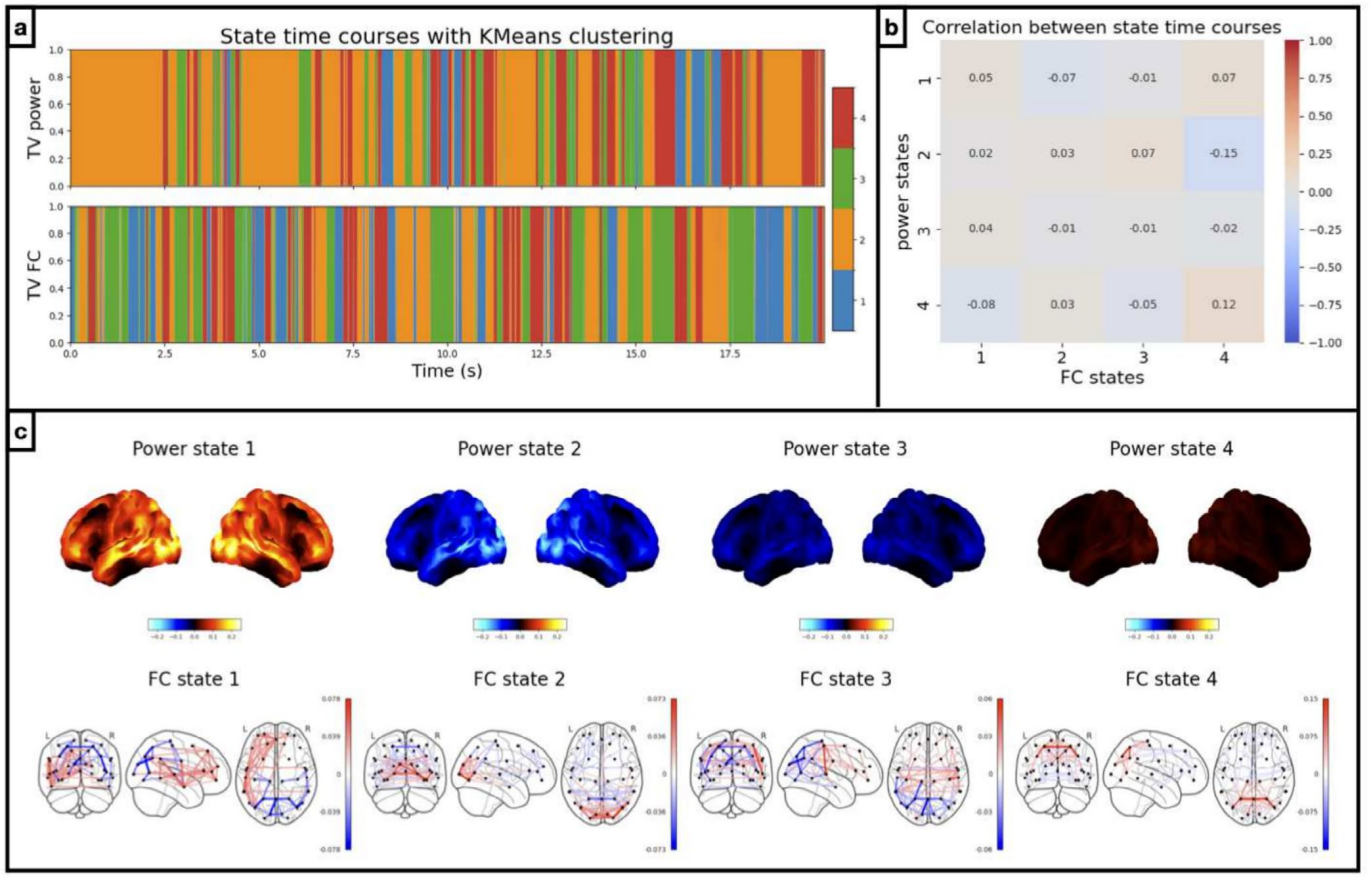
Sliding window approach reveals distinct power and FC dynamics. Results from amplitude time courses (1–45 Hz) of 38 brain regions obtained from the MEGUK‐38 resting‐state data. (a) K‐means state time courses for TV variance (i.e., power) (top row) and correlation (i.e., FC) (bottom row), using 2s sliding windows. Different colours indicate activation of different brain states and only the first 20 s of the first subject is shown. (b) Correlation between power (y‐axis) and FC state time courses (x‐axis). (c) Power maps of each K‐Means state are plotted on the top row. Red areas indicate brain areas with higher power (and blue areas lower power) than the average across states. FC maps are plotted on the bottom row. Red edges indicate positive correlations (and blue edges negative correlations) between brain areas.

Firstly, we apply the well‐established sliding window approach followed by K‐means clustering to identify two separate dynamics according to TV power (variance) and TV FC (correlation) respectively (See Section 2.3, Allen et al. 2014) from amplitude time courses (1–45 Hz) of 38 brain regions obtained from MEGUK‐38 resting‐state data. Figure 1a illustrates the first 20 s of the state time courses (K‐means cluster assignments) for the first subject in the MEGUK‐38 resting‐state data. Qualitatively, we can see that TV power and FC do not follow the same dynamics, and the correlation between the two time courses is low, as shown in Figure 1b. Furthermore, each of the two state time courses corresponds to reasonable power and FC maps, respectively, demonstrated in Figure 1c.

While Figure 1 provides preliminary evidence that power and FC have distinct dynamics, we do not know if this is an artefact of the choice of arbitrary hyperparameters (e.g., window size) or the inability of sliding window approaches to automatically adapt over time to fast dynamics (Appendix Section C.2 demonstrates this on simulated data). We instead need a method that can infer multiple dynamics in an adaptive, data‐driven way. This motivates using our new approach, Multi‐dynamic Network Modes (M‐DyNeMo).

### 3.2 Power and FC Have Similar Spatial Patterns but Distinct Dynamics

**FIGURE 2:**
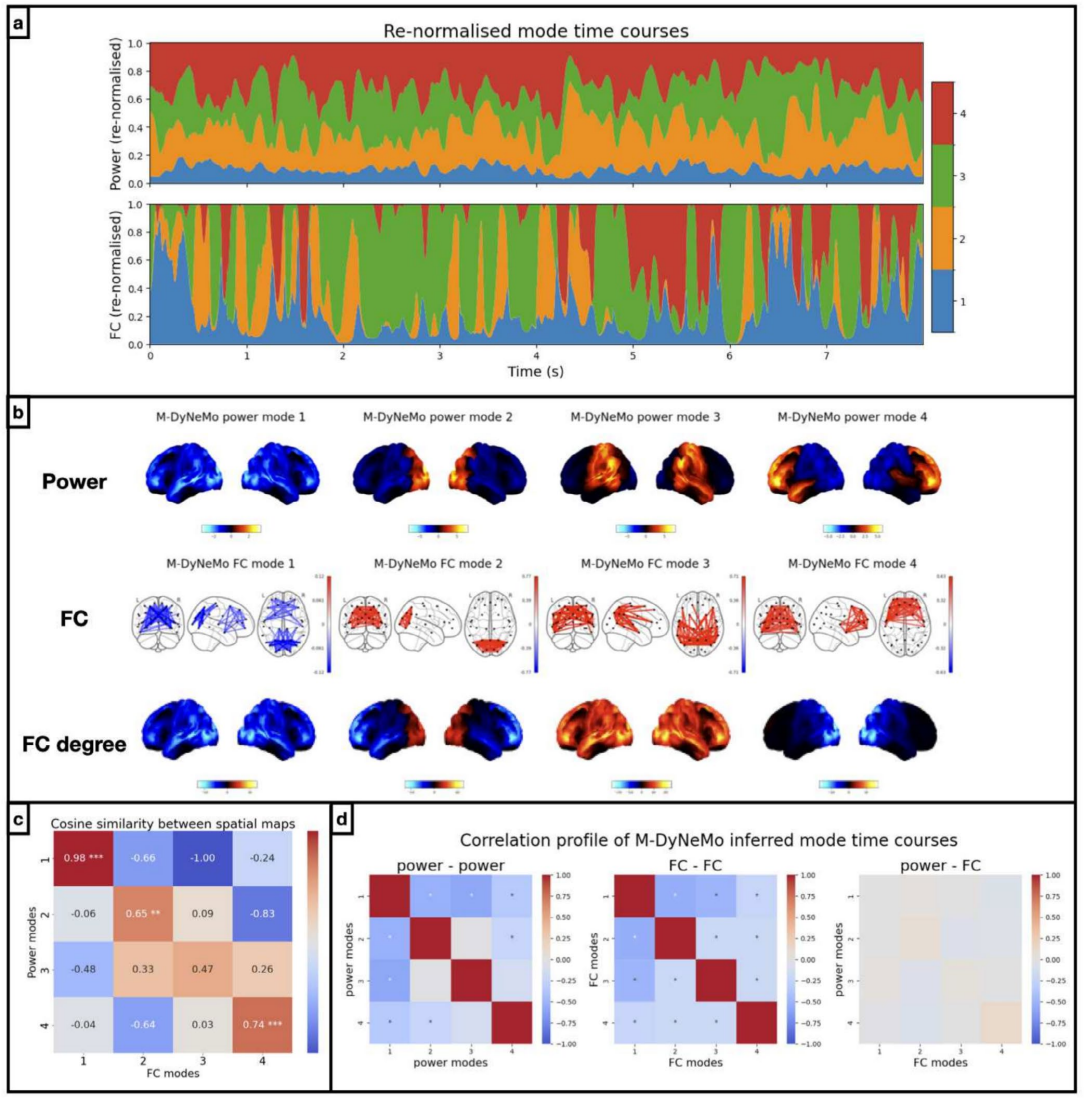
Power and FC have similar spatial patterns but distinct dynamics. Results of running M‐DyNeMo on the amplitude time courses (1–45 Hz) of 38 brain regions obtained from the MEGUK‐38 resting‐state data. (a) Inferred mode time courses (renormalised) for power, α (top) and FC, β (bottom). (b) Top row shows power spatial modes where red areas show higher (blue areas show lower) activation than average across modes. Middle row shows FC spatial modes where red edges show positive (and blue edges show negative) connectivity. Bottom row shows the FC degree maps where red areas show higher (blue areas show lower) node degree (i.e., centrality) than average across modes. (c) Pairwise cosine similarity between power and FC degree spatial maps. Diagonal entries with significantly large values are annotated with asterisks depending on the *p*‐values. (d) Correlation profile of mode time courses for within power modes (left), within FC modes (middle) and between power and FC modes. Correlations significantly (at 5% significance level) differ from zero under a maximum statistic permutation test are marked with an asterisk.

First, we trained M‐DyNeMo on the amplitude time courses (1–45 Hz) of 38 brain regions obtained from MEGUK‐38 resting‐state data. Figure 2a shows the renormalised mode time courses for the first 8 s of the first subject in this dataset (See Appendix Section D.1 for details on renormalisation). We see qualitatively that mode time courses for power (α) and for FC (β) possess very different characteristics, in the sense that β is much more binarised than α.

Power and FC networks for each mode are shown in Figure 2b. These networks resembled those found in multiple previous studies (Baker et al. 2014; Gohil et al. 2022, 2024b; Vidaurre et al. 2016). For a clearer visual comparison, we also show the FC degree maps, each of which plots the sum of correlations for each node, relative to the average across modes. Note that the ordering of spatial modes found by the model is arbitrary; hence, the network maps shown in this figure have been reordered post hoc to match the spatial patterns of activity in power and FC for each pair of modes. In particular, power and FC mode 1 show below‐average activities across the brain. We believe their presence is due to the sum‐to‐one constraint in the generative model of the mode time courses, that is, these are ‘background’ networks that are active when other networks are not active. This is also observed and discussed in the original DyNeMo paper (Gohil et al. 2022). Even though there is no requirement for them to be similar, the power and the FC spatial maps have highly corresponding spatial patterns. Specifically, there are significantly high cosine similarities between power and FC degree maps for mode pairs 1, 2, and 4 under a maximum statistic permutation test (see Appendix Section D.2 for details), illustrated by the diagonal entries in Figure 2c.

Although we identify power and FC modes that show activity in the same regions, their corresponding mode time courses (dynamics, α vs. β) are not significantly correlated (under a maximum statistic permutation test across subjects), as demonstrated in Figure 2d, plotted on the right, suggesting time points with high power in a particular region may not necessarily also have high FC in the same region. The correlations within the power mode dynamics (α, left) and within the FC mode dynamics (β, middle) are mostly negative, indicating when a mode activates the others deactivate.

In summary, Figure 2 shows that power and FC have similar spatial patterns but distinct dynamics. However, one concern is that the distinct dynamics might be enforced by the assumptions made by M‐DyNeMo. To mitigate this concern, we used simulations in Appendix Section C.3 to show that if the data are generated using a single, shared dynamic for power and FC, then M‐DyNeMo does not incorrectly infer distinct dynamics.

### 3.3 Single Dynamic DyNeMo is Dominated by Time‐Varying Power

**FIGURE 3:**
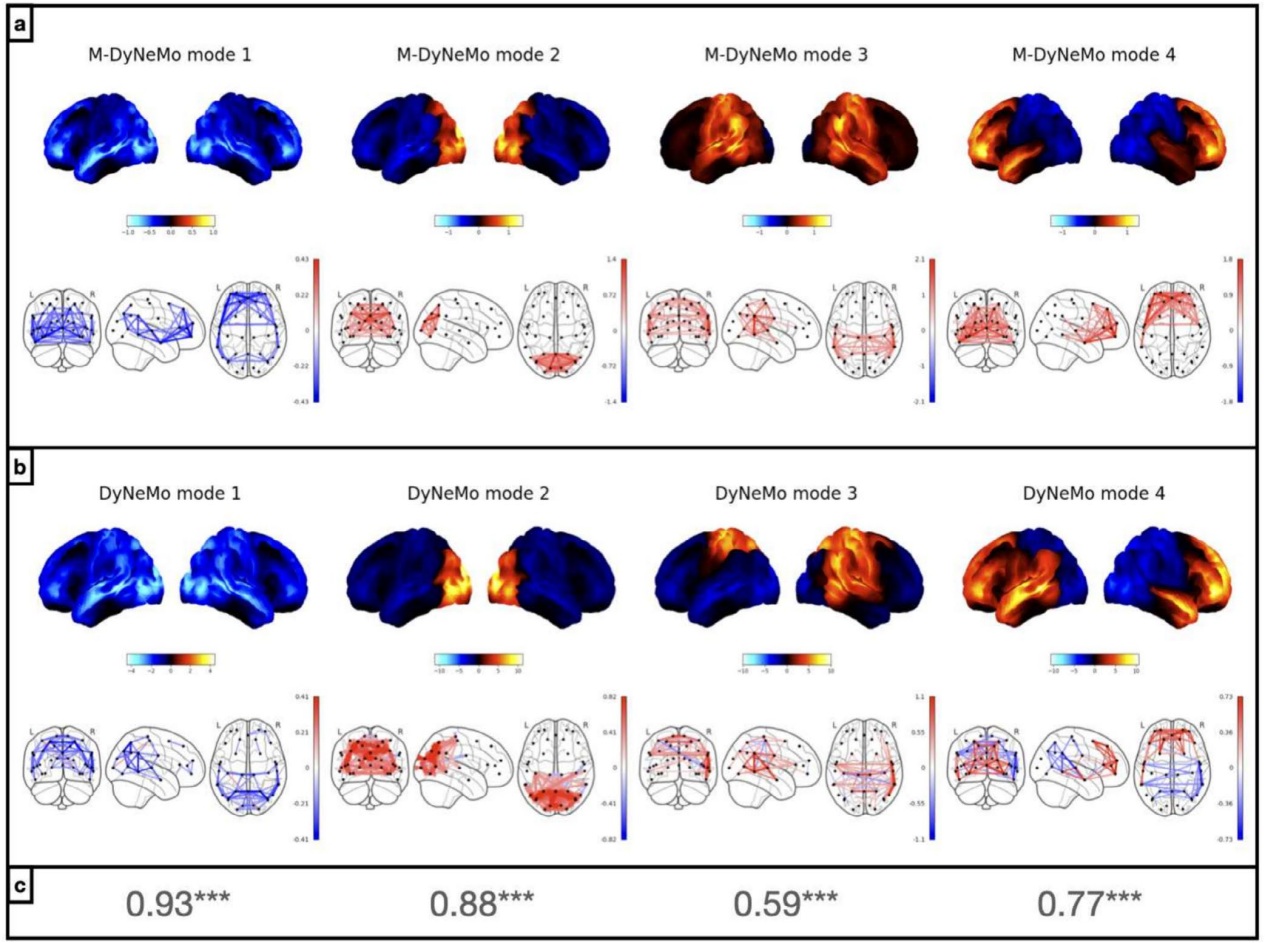
Single dynamic DyNeMo is dominated by time‐varying power. Results from amplitude time courses (1–45 Hz) of 38 brain regions obtained from MEGUK‐38 resting‐state data. (a) Power (top row) and FC (covariance, bottom row) network maps given by recalculating time varying covariances on M‐DyNeMo inferred power mode time course α. (b) Power (top row) and FC (covariance, bottom row) maps given by DyNeMo. (c) Cosine similarity between recalculated covariances on M‐DyNeMo inferred power mode time course α and DyNeMo inferred covariances for each pair of modes.

**FIGURE E.1:**
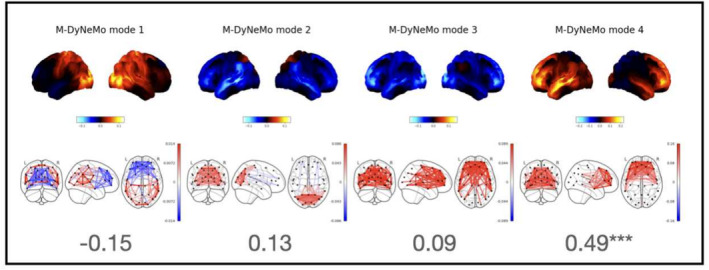
Single dynamic DyNeMo is dominated by time‐varying power. Results from amplitude time courses (1–45 Hz) of 38 brain regions obtained from MEGUK‐38 resting‐state data. Power (top row) and FC (covariance, bottom row) network maps given by recalculated networks using M‐DyNeMo inferred FC mode time course β. The cosine similarity is also shown for each pair of modes between the recalculated covariances on M‐DyNeMo inferred FC mode time course β and standard DyNeMo inferred covariances.

Given the evidence that there are distinct dynamics, we were interested to learn which of the dynamics dominate the decomposition when only a single dynamic is allowed, as is the case in standard DyNeMo. To investigate this we assumed that the power and FC share a single dynamic given by the power mode time course α, extracted from a trained M‐DyNeMo, and then recalculated the power and FC for each mode. The resulting networks are shown in Figure 3a. Comparing this with the networks inferred by standard DyNeMo on the same amplitude time courses (1–45 Hz) of 38 brain regions from the MEGUK‐38 resting‐state data, shown in Figure 3b, we can see that the power mode time course $\left(\alpha \right)$ inferred by M‐DyNeMo contains similar information as the mode time course inferred by standard DyNeMo. Quantitatively, for each pair of modes, there is a significantly large cosine similarity (under a maximum statistic permutation test) between the recalculated networks using M‐DyNeMo inferred power mode time course α and the inferred networks given by standard DyNeMo. This suggests the description provided by standard DyNeMo is dominated by the dynamics in power rather than FC. Furthermore, by recalculating networks using M‐DyNeMo inferred FC mode time course β, we show in Appendix Figure E.1 that the FC dynamics given by M‐DyNeMo contains different information as the mode time course inferred by standard DyNeMo.

### 3.4 M‐DyNeMo Inferred Networks Are Reproducible Across Subsets of Data

**FIGURE 4:**
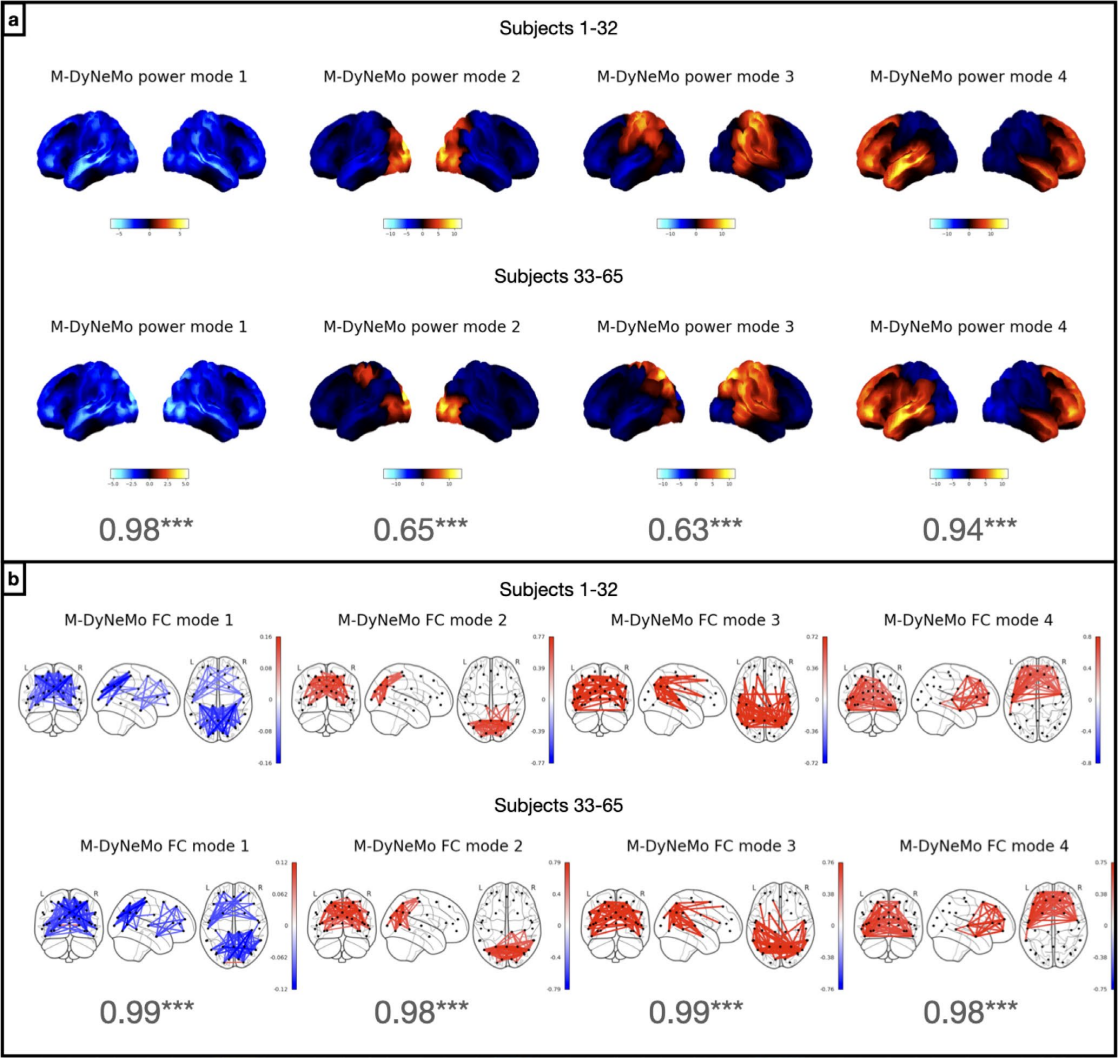
M‐DyNeMo inferred networks are reproducible across halves. Results from amplitude time courses (1–45 Hz) of 38 brain regions obtained from MEGUK‐38 resting‐state data. (a) Power spatial modes given by training M‐DyNeMo on Subjects 1–32 (top row) and Subjects 33–65 (bottom row) independently. The cosine similarity between power maps from each half is also shown for each pair of modes. (b) FC spatial modes given by training M‐DyNeMo on Subjects 1–32 (top row) and Subjects 33–65 (bottom row) independently. The ordering of both power and FC modes are matched with cosine similarities between networks from each half, which are shown in the figure for both power and FC networks.

To evaluate the reproducibility and robustness of the model, we train M‐DyNeMo on different subsets of subjects of the MEGUK‐38 resting‐state dataset. We split 65 subjects of this dataset into two halves where the first half contains Subjects 1–32 and the second half contains Subjects 33–65. M‐DyNeMo is trained independently on each half. Figure 4a,b, shows that network maps inferred by M‐DyNeMo on each half, with modes matched using cosine similarity, which is also shown in the figure for each pair of modes. In general, we see the same networks in each half with significantly large cosine similarity under a maximum statistic permutation test. There are only slight differences in the spatial pattern of activity in power modes 2 and 3.

### 3.5 Power and FC Dynamics Show Different Timings in Evoked Network Response

In Section 3.2, we saw M‐DyNeMo infers distinct dynamics (i.e., uncorrelated mode time courses) for power and FC using the amplitude time courses (1–45 Hz) from resting‐state MEG data. We now turn to task MEG data and ask the question: Do α and β respond differently to tasks? We perform this investigation with the Wakeman–Henson dataset, where participants are presented with images of famous, unfamiliar, or scrambled faces. This allows us to study how the mode time courses respond to visual stimuli as well as the difference in responses to different visual stimuli.

**FIGURE 5:**
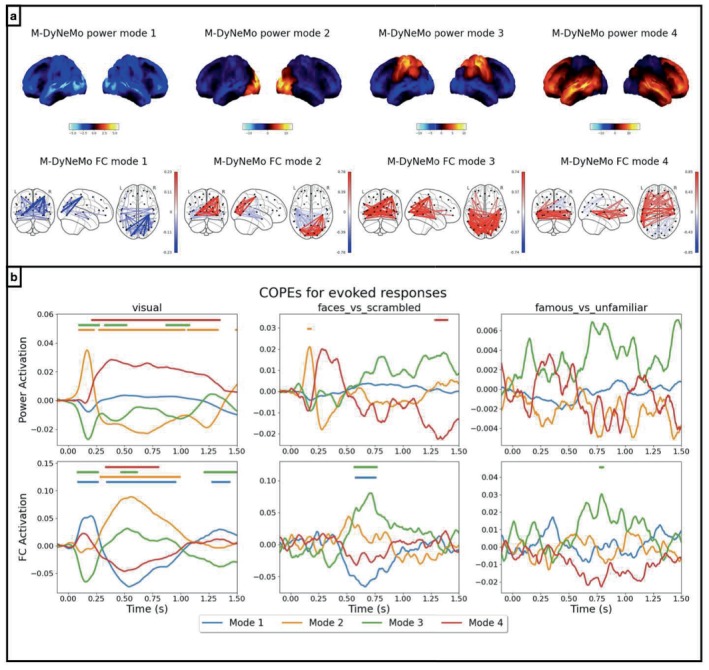
Evoked responses have different timings in different mode time courses. Results from amplitude time courses (1–45 Hz) of 38 brain regions obtained from Wakeman‐Henson data. (a) Power spatial modes are shown on the top row and FC spatial modes are shown on the bottom row. (b) Evoked responses for renormalised α (top row) and β (bottom row) for different contrasts on different columns, including the average visual response (left), the difference between faces and scrambled faces (middle), and the difference between famous and unfamiliar faces (right). Here, different colours represent baseline corrected evoked responses for different modes as contribution to either the overall variance (for α) or the overall correlation (for β). Periods with significant (de‐)activation are indicated with a bar for each mode.

The network plots are shown in Figure 5a, which are comparable to those inferred from MEGUK‐38 resting‐state data, shown in Figure 2b. In Figure 5b, the evoked network responses are shown for different contrasts, including the average of all visual stimuli, the difference between faces (including both famous and unfamiliar faces) and scrambled faces, and the difference between famous and unfamiliar faces.

#### 3.5.1 Power Dynamics

We focus on the top row of Figure 5b first. Similar to the analysis performed in (Gohil et al. 2024b), a two‐level general linear model (GLM, Winkler et al. 2014]) is used for computing evoked network responses, where an evoked response analysis is carried out on each of the task‐epoched M‐DyNeMo mode time courses. Note that this is carried out *after* M‐DyNeMo is trained, that is, M‐DyNeMo has no knowledge of the task timings. A maximum statistic permutation test is used to identify periods of significant responses.

We see in the top row of Figure 5b that evoked network responses for power, α, are broadly consistent with those given by training standard DyNeMo on the same data (see Appendix Figure E.3). For example, in the average visual response, there is an immediate activation of the visual network (α mode 2) followed by an activation of the frontal network (α mode 4) and a deactivation of the visual network. In the difference between unscrambled faces (famous plus unfamiliar) and scrambled faces, there is an activation of the visual network and a delayed deactivation of the frontotemporal network. The only difference is that there is no activation of the suppressed network (α mode 1) at around 600 ms after the onset like in the analysis of standard DyNeMo. There are no significant differences when we look at the responses to famous and unfamiliar faces.

#### 3.5.2 FC Dynamics

We now draw our attention to the bottom row of Figure 5b where the evoked responses of the FC network mode dynamics, β, are illustrated. For the average visual response (left), we see an immediate activation of Mode 1 and deactivation of Mode 3, followed by a delayed and persistent activation of Mode 2 as well as a deactivation of the Mode 1 and Mode 4. Looking at the differences in response to faces and scrambled faces, there is only a delayed activation of Mode 3 and a deactivation of the Mode 1. Finally, there is a short‐lived activation of the Mode 3 when comparing responses to famous and unfamiliar faces.

### 3.6 The Coupling Between Power and FC Dynamics is Modulated by Task

As shown in Figure 5, there is evidence of different timings in evoked network responses for power and FC dynamics. This motivated us to investigate whether the extent to which there is coupling between power dynamics and FC dynamics is different between task and rest. To this end, we train M‐DyNeMo on a new dataset, MEGUK‐52, which contains 63 subjects who have both resting‐state and visual task recordings, totalling 126 sessions.

**FIGURE 6:**
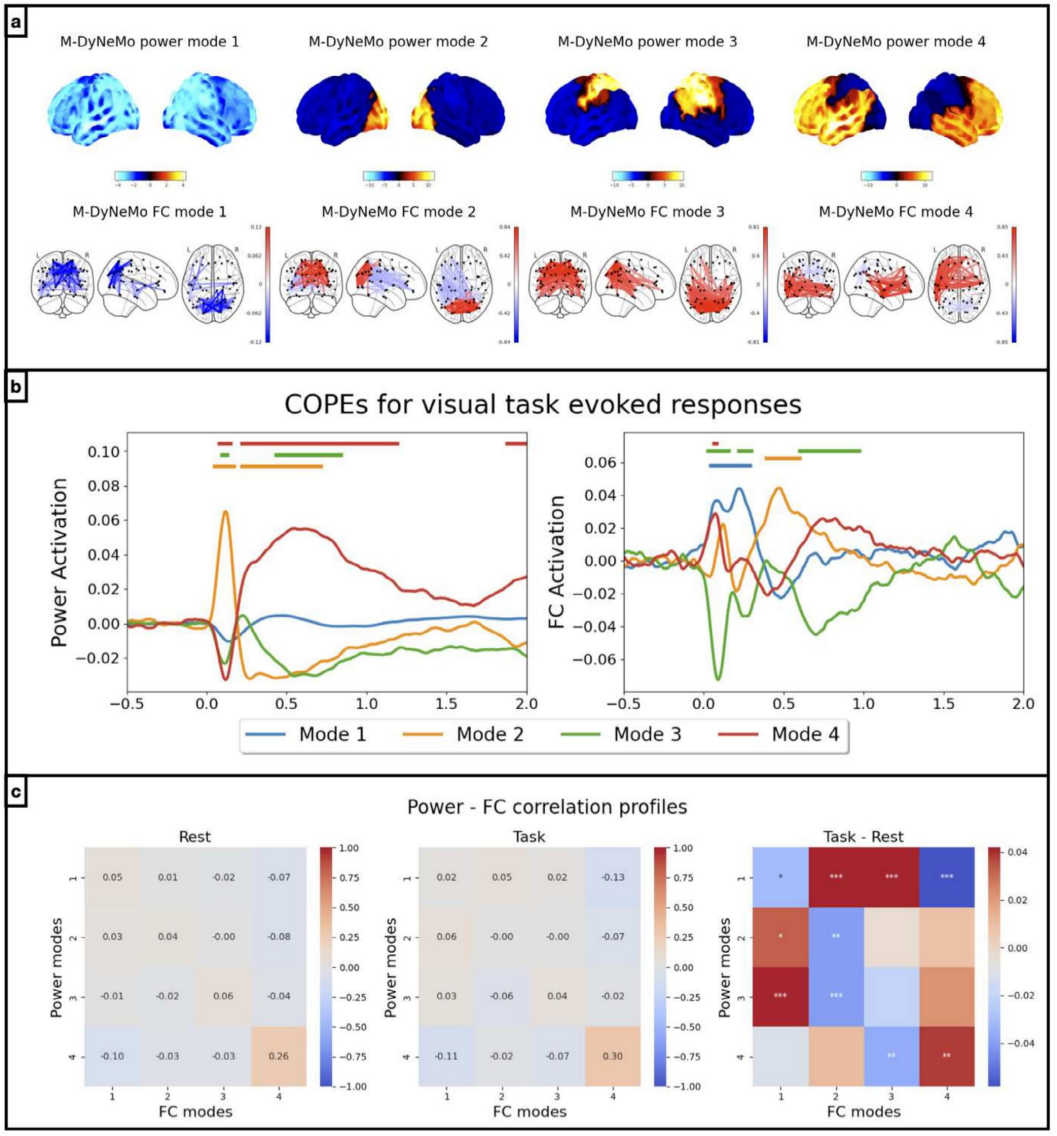
Coupling between α and β is modulated by task stimuli. Results from amplitude time courses (1–45 Hz) of 52 brain regions obtained from MEGUK‐52 data. (a) Power (top row) and FC (bottom row) spatial modes are plotted. (b) Evoked responses (baseline corrected) for renormalised α (left) and β (right) to visual stimuli. Different colours show evoked responses for different modes. (c) Correlations between α and β during rest (left) and during task (middle) are shown. The right plot illustrates difference between the correlation profiles where significant differences are marked with asterisks.

Figure 6a shows the inferred networks, which are broadly consistent with those inferred on the same dataset, using a different parcellation compared to in Section 3.2 (MEGUK‐38 resting‐state data) and on a different dataset than Section 3.4 (Wakeman–Henson—which only contains visual task data and on a different set of subjects). This again demonstrates the reproducibility of M‐DyNeMo on different datasets and parcellations.

The evoked network responses to visual stimuli, shown in Figure 6b, which is consistent with the findings in Section 3.4. Figure 6c shows the correlations between the power (α) and FC (β) dynamics during rest (left), those during epochs of 0.5 s before and 2 s after the visual stimuli (middle), as well as the difference in correlations between task and resting state. In particular, from rest to task, we see increased correlation of α Mode 2 with β Mode 1 and decreased correlation with β Mode 2, which is consistent with the immediate activation of α Mode 2 and β Mode 1, and the delayed activation of β Mode 2 in Figure 6b. It is worth noting that the sliding window approach on this dataset also provides evidence for the difference in the coupling of the two dynamics (See Appendix Figure E.4).

## 4 Discussion

With resting‐state MEG data, we found preliminary evidence for the existence of distinct power and FC dynamics using a standard sliding window approach. However, this approach lacks the ability to adapt to transient dynamics at unknown time scales in electrophysiological data. We demonstrated the deficiency of sliding window approaches to do this using simulated data in Appendix Section C.2. Therefore, we proposed a new generative model approach that automatically tunes to the required time scales based on the data and that can incorporate multiple dynamics: M‐DyNeMo.

Using M‐DyNeMo, we show that while they have similar spatial patterns, the two mode time courses, one for power and one for FC, possess very different characteristics, and there is no significant correlation between them, that is, they are decoupled. The same property can also be found in two other datasets (see Appendix Figure E.5). It should be noted that there is no constraint imposed by the generative model or inference framework in M‐DyNeMo on the dynamics. It is solely the data itself that decides the dynamics are decoupled, and the FC dynamics are more binarised than the power dynamics. We believe that the information driving the M‐DyNeMo inferred mode time courses is similar to that driving the time courses in other methods such as ICA and K‐means clustering, but M‐DyNeMo offers additional temporal regularisation through the LSTM, which learns the temporal structure during training. Hence, the mode time courses inferred by M‐DyNeMo can be thought to be ‘temporal regularised’.

Aside from resting‐state data, we also explore the additional insights M‐DyNeMo provides on task data. In Section 3.4, we show that the two‐mode time courses respond differently to visual stimuli. Moreover, we provide evidence that coupling between power and FC dynamics is modulated by stimuli. This could be related to the findings in (Barttfeld et al. 2015) that complexity in FC dynamics is inversely proportional to the level of consciousness. Overall, our results demonstrate how M‐DyNeMo has opened a new door to studying the relationship between cognition and dynamic FC.

**FIGURE E.2:**
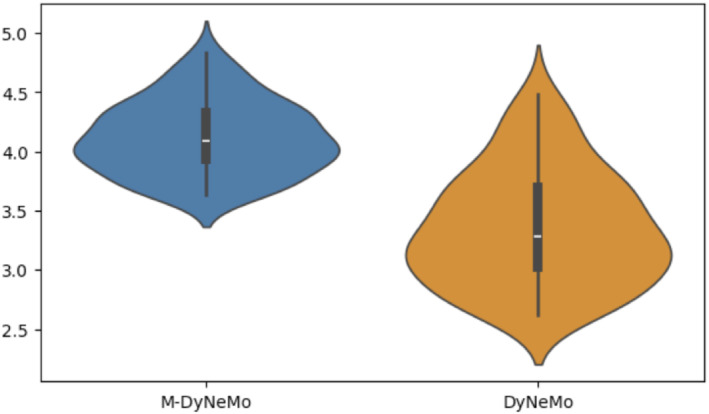
**M**‐DyNeMo reconstructed time varying covariances show greater fluctuation over time. Results from amplitude time courses (1–45 Hz) of 38 brain regions obtained from MEGUK‐38 resting‐state data. For M‐DyNeMo and DyNeMo, the y‐axis shows the variability of model reconstructed time varying covariances in terms of Riemannian distance of time varying covariances from the mean covariance for each subject.

**FIGURE E.3:**
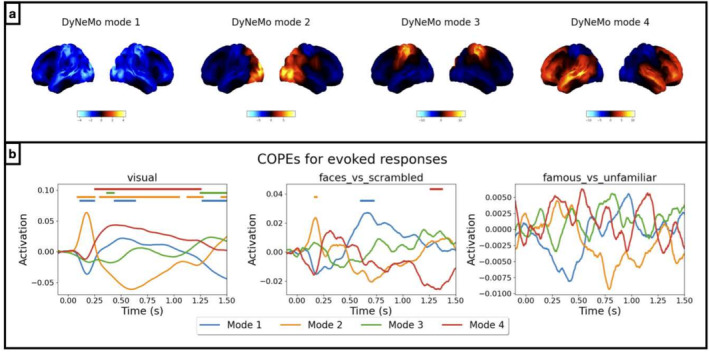
Evoked responses given by DyNeMo. Results from amplitude time courses (1–45 Hz) of 38 brain regions obtained from Wakeman–Henson data. (a) Power maps for different modes given by DyNeMo. (b) Evoked response analysis on DyNeMo‐inferred mode time course. Different colours show baseline corrected evoked response (contribution to overall variance) of different modes.

**FIGURE E.4:**
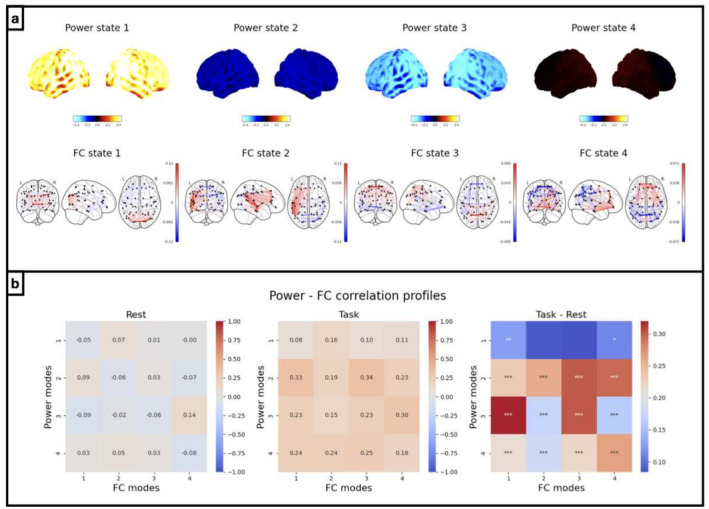
Sliding window approach reveals evidence of different correlation profiles during task versus during rest. Results from amplitude time courses (1–45 Hz) of 52 brain regions obtained from MEGUK‐52 data. (a) Power (top row) and FC (bottom row) maps for each mode of the two state time courses. (b) Correlations within α (left) and within β (middle) are shown. The right plot shows the differences in correlations during task versus during rest. Correlations that significantly differ from zero are marked with asterisks depending on the *p*‐values.

**FIGURE E.5:**
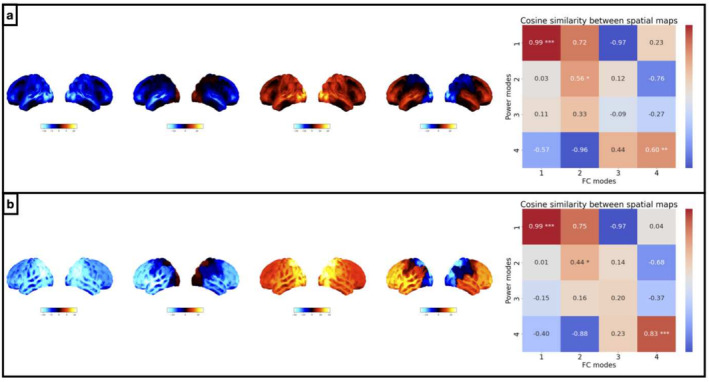
Additional FC degree spatial maps. Results from amplitude time courses (1–45 Hz) of 38 brain regions obtained from Wakeman‐Henson data and of 52 brain regions obtained from MEGUK‐52 data. (a) FC degree spatial maps on the Wakeman‐Henson dataset (left) and the cosine similarity between power maps and FC degree maps for each pair of power and FC modes (right). (b) FC degree spatial maps on the MEGUK‐52 dataset (left) and the cosine similarity between power maps and FC degree maps for each pair of power and FC modes (right).

On simulated data where power and FC follow separate dynamics, we can show M‐DyNeMo performs more accurate inference than DyNeMo (Appendix C.2). Furthermore, in Section 3.3, we showed that standard, single‐dynamic DyNeMo tends to ignore the FC dynamics in real data and is dominated by the power dynamics. This is consistent with the idea that the signal from time‐varying FC is weaker in real data than that from power, which is perhaps to be expected given that power is easier to estimate in general. If we look at the fluctuation of the reconstructed time‐varying covariances from both M‐DyNeMo and standard DyNeMo (Appendix Figure E.2), we can see that the shared‐dynamics constraint of standard DyNeMo causes lower variability in FC, which is consistent with the findings of applying multi‐dynamic modelling to fMRI data (Pervaiz et al. 2022). Using standard single‐dynamic DyNeMo, consistent differences have previously been found in mode time courses between young and old participants (Gohil et al. 2024a). Therefore, a future direction is to see if the FC mode time courses, which to date will have been ignored due to its relatively low signal in real data, can provide more understanding into ageing and distinctions between healthy and diseased brains.

In Appendix Section C.3, we showed that if the underlying data is generated by a single dynamic process, then M‐DyNeMo does not enforce distinct dynamics for FC and power, and correctly infers a single, shared set of dynamics. It is worth noting that although data are simulated using the generative model of a hidden Markov model, where time courses are binarised, M‐DyNeMo is still capable of learning from the data and inferring binarised time courses, without a constraint being posed on its generative model. We have also explored using an edge time series technique proposed by (Faskowitz et al. 2020) on the simulated dataset. However, we find it gives noisy estimates of FC and fails to capture the transient temporal changes accurately.

Finding the best model hyperparameters, for example, the number of clusters in K‐Means clustering, the number of states in the HMM, is a long‐standing challenge in unsupervised learning models (Baker et al. 2014; Gohil et al. 2022; Vidaurre et al. 2016), and M‐DyNeMo is not an exception. Due to the increased flexibility and complexity of the model, M‐DyNeMo presents significant challenges regarding the stability of training and the reproducibility of results. The tricks in Section 2.4.1 and Appendix Section B.2 were introduced to help with this issue. In this work, we chose both the number of power and FC modes to be 4, which is low enough for reproducibility and high enough for meaningful results. In Figure 4, we assessed the reproducibility of M‐DyNeMo with the split‐half reproducibility, and we consider the model to be stable when the best model (based on training loss) out of 10 independent runs of each half gives similar (measured by cosine similarity) spatial maps. We acknowledge that the reproducibility values in Figure 4 will likely deteriorate with a larger number of modes, but this does not invalidate the reproducibility of the model under the hyperparameters chosen in this paper. A formal analysis of the effect of both the number of power and FC modes, and finding a systematic way to choose them, is no doubt an important work in the future.

In this paper, we used amplitude envelope time courses (1‐45 Hz) from MEG data, and correspondingly used correlation between amplitude envelopes as a measure of FC. However, this ignores more detailed spectral information in the MEG signal. For example, the phase‐locking between brain regions (coherence) has recently gained increasing interest as a measure of FC in electrophysiology data. (Vidaurre et al. 2016) introduced a method called *time‐delayed embedding* for including spectral information into the covariance matrix of the time‐delay embedded data. However, it is not trivial to decompose this covariance matrix into parts that correspond to power and parts that correspond to FC, similar to what has been done in Section 2.4. The additional PCA step for reducing dimensionality complicates the modelling even further. Another direction is to filter the data into different frequency bands and include them as additional channels of the training data. This will allow us to investigate the uncoupling of dynamics in different frequency bins and potentially dynamics of cross‐frequency interactions.

## 5 Conclusions

We proposed a new method for inferring potentially separate power and FC dynamics. We show that the proposed model is robust and inferred networks are reproducible across subjects, datasets, and parcellations. We use this to show that power and FC have similar spatial patterns, but distinct dynamics, in ongoing MEG brain activity. Furthermore, we show that the two types of dynamics respond differently to visual stimuli in MEG task data, and that the extent to which there is coupling between the two types of dynamics is modulated by the task.

## Ethics Statement

The Wakemen–Henson dataset (Wakeman and Henson 2015) was approved by the Cambridge University Psychological Ethics Committee. Written informed consent was obtained from participants. As part of the UK MEG Partnership, the MEGUK dataset was collected at the University of Nottingham. All participants gave written informed consent, and ethical approval was granted by the University of Nottingham Medical School Research Ethics Committee.

## Conflicts of Interest

Board member is co‐author: Mark Woolrich is a member of the HBM Editorial Board and co‐author of this article.

## Data and Code Availability Statement

Data used are publicly available. Availability of MEGUK is at the official site.

https://meguk.ac.uk/database/ and for Wakeman–Henson, we refer the readers to the original paper (Wakeman and Henson 2015). Source code for M‐DyNeMo is available in the *osl‐dynamics* toolbox (Gohil et al. 2024a) and scripts to reproduce results in this manuscript are available at:

https://github.com/OHBA‐analysis/Huang2024_EvidenceForUncoupledDynamics.

## Figures and Tables

**FIGURE C.1:**
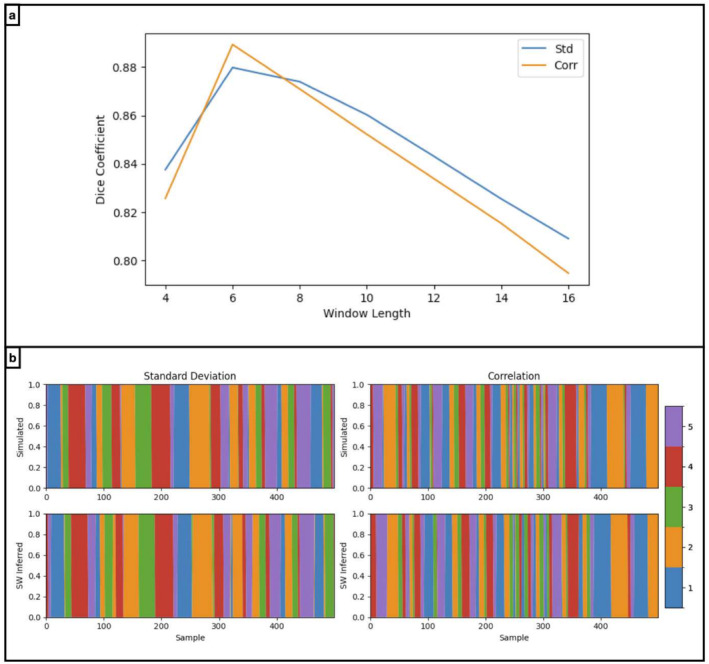
Sliding window approach fails to infer ground truth state time courses. Results on Simulation 1 dataset. (a) Dice coefficient of state time courses inferred by sliding window approach using different window lengths. (b) The simulated (top row) and sliding window approach inferred (bottom row, with window size of six samples) time courses. A segment of 500 samples containing short state transitions is shown.

**FIGURE C.2:**
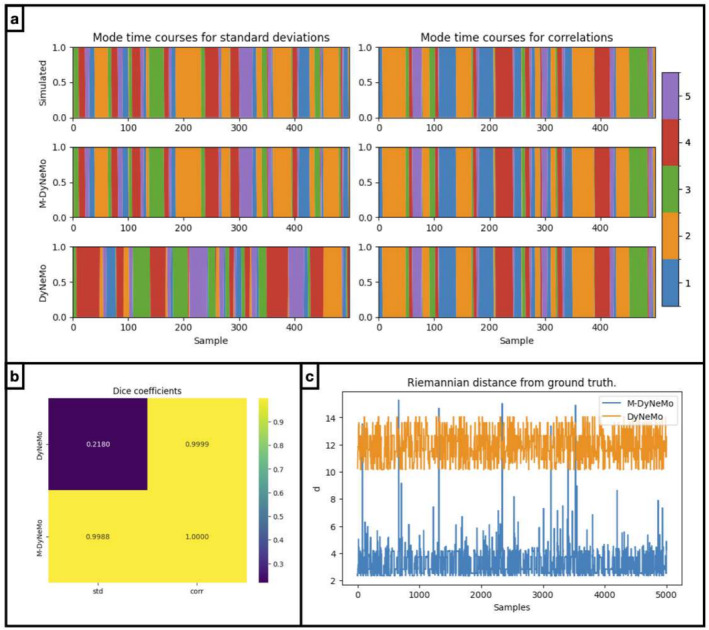
M‐DyNeMo infers time courses more accurately than DyNeMo when data has multiple dynamics. Results on Simulation 1 dataset. (a) The simulated (top row), M‐DyNeMo inferred (middle row) and DyNeMo inferred (bottom row) time courses for time‐varying standard deviations (left column) and correlations (right column). Here, different colours indicate activation of different states and only the first 500 samples are plotted. (b) DICE coefficients of DyNeMo and M‐DyNeMo inferred time courses compared to the simulated ground truth. (c) The reconstruction error (Riemannian distance) of the first 500 samples of time varying covariances is plotted against sample on the x‐axis. The reconstruction error of DyNeMo is plotted in orange and that of M‐DyNeMo is plotted in blue.

**FIGURE C.3:**
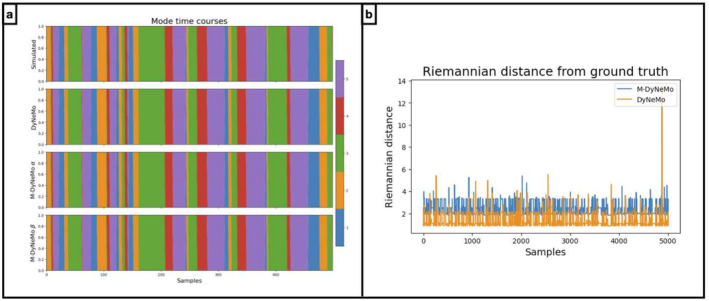
M‐DyNeMo infers accurately on data with single dynamic. Results on Simulation 2 dataset. (a) From top to bottom row are the ground truth simulated, the DyNeMo inferred, and the two ‘separate’ M‐DyNeMo inferred mode time courses. Here, different colours indicate activation of different states and only the first 500 samples are plotted. (b) The reconstruction error (Riemannian distance) of the first 5000 samples of time‐varying covariances is plotted against sample on the x‐axis. The reconstruction error of DyNeMo is plotted in orange and that of M‐DyNeMo is plotted in blue.

## Data Availability

The data that support the findings of this study are available in Huang2024_EvidenceForUncoupledDynamics at https://github.com/OHBA‐analysis/Huang2024_EvidenceForUncoupledDynamics. These data were derived from the following resources available in the public domain: MEGUKI, https://meguk.ac.uk/database/‐A multi‐subject, multi‐modal human neuroimaging dataset, https://openfmri.org/dataset/ds000117/.

## Appendix A Generative Model of M‐DyNeMo

Here, we give an illustration of the generative model of M‐DyNeMo in Figure A.1.

## Appendix B Model Training Details

### Learning Correlation Matrices

A square matrix $R\in R^{n\times n}$ is a correlation matrix if and only if it is a covariance matrix with diagonal of 1. Similar to learning a covariance matrix, we cannot learn the entries of a correlation matrix directly and need to parameterise the matrix with a vector using a bijection. Similar to learning a covariance matrix, we resort to learning the Cholesky decomposition of a correlation $R=LL^{T}$, where the k,l element $L_{\mathrm{kl}}$ satisfies:

(B1)
$$L_{\mathrm{kl}}=\left\{\begin{array}{cc} \;0 & \text{if}\;k<l,\\ M_{\mathrm{kl}}/w_{k} & \text{if}\;k>l,\\ \;1/w_{k} & \text{if}\;k=l, \end{array}\right.$$

where $M_{\mathrm{kl}}$ is the k,l entry of a lower triangular matrix M and w is a vector such that its *k*‐th element is $w_{k}=\sqrt{1+\sum_{l=0}^{k-1}M_{\mathrm{kl}}^{2}}$. It is easy to check that R constructed in this way is a legitimate correlation matrix and during training, we learn the vector of lower triangular entries of M.

### Extra Tricks for Model Training

Some extra tricks were used to help fast and stable convergence of M‐DyNeMo:

- **KL annealing**: M‐DyNeMo can be thought of as a special case of the variational autoencoder (VAE) and optimising a VAE with RNN is challenging. In most runs, the KL term dominates and the model consistently sets the variational distribution close to the prior distribution of the latent variables, yielding very small KL term (Bowman et al. 2015). The solution is surprisingly simple and effective: annealing the KL term. The idea is to train the model with a modified loss function where the KL term is multiplied by an annealing factor κ that starts at 0 and gradually increases to 1 as the training progresses, that is.
  (B2)
  $${ℒ}_{n}=-\mathrm{LL}+\kappa \mathrm{KL}.$$
  This allows the model to first learn a good encoder before tuning the variational distribution to match the prior distribution.
- **Multistart initialisation**: The objective function is highly nonconvex and the model can end up in different local minima depending on the initialisation. To mitigate this, a multi‐start initialisation strategy is used. At each initialisation, the model parameters are initialised randomly and the model is trained for a small number of epochs. This is repeated multiple times and the model with the lowest loss is chosen for further training.
- **Learning rate scheduling**: A learning rate scheduler is used to exponentially reduce the learning rate as the training progresses. This helps the model to take large steps in the beginning for faster convergence and smaller steps towards the end for fine‐tuning. In practice, we only start decreasing the learning rate after the KL annealing phase.
- **Initialisation for** $R_{j}$: We find in practice that without a ‘good’ initialisation of the correlation matrices, the convergence of the model is extremely slow. To help with this, we initialise the correlation matrices with ‘realistic’ ones obtained from the data. Specifically, we use a sliding window approach to calculate the time‐varying correlation matrices and used K‐means clustering to identify $J^{2}$ clusters of time‐varying correlation matrices. We then initialise the correlation matrices with the cluster centroids. We find that this helps the model to converge to a lower loss consistently.

## Appendix C Simulation Results

In this section, we describe how we use simulation to validate M‐DyNeMo and investigate potential benefits of multidynamic modelling.

### Simulated Data

We simulate data with the Hidden Markov Model (HMM) generative model. We prespecify the number of states $J^{1}=J^{2}=J$, the transition probability A and randomly simulate the observation model parameters $\theta_{\mathrm{obs}}$, which are then used to simulate the observed data. Two simulation datasets are used:

- **Simulation 1**. Here, we simulate data with multiple dynamics using $J=5,N_{c}=40,T=25600$. The transition probability matrix is
  (C1)
  $$A=\left[\begin{array}{ccccc} 0.9 & 0.1 & 0 & 0 & 0\\ 0 & 0.9 & 0.1 & 0 & 0\\ 0 & 0 & 0.9 & 0.1 & 0\\ 0 & 0 & 0 & 0.9 & 0.1\\ 0.1 & 0 & 0 & 0 & 0.9 \end{array}\right].$$
- **Simulation 2**. Here, data are simulated with the same setting as Simulation 1 except that $E_{j},R_{j}$ follow the same underlying temporal dynamics, that is, they depend on the same underlying Markov chain of states.

### Simulation 1: M‐DyNeMo Performs More Accurate Inference on Data With Multiple Dynamics

Here, we aim to see if M‐DyNeMo can perform inference more accurately than the traditional sliding window approach and DyNeMo, given the data have multiple dynamics. We focus on the first simulated data in Section C.1 and follow the procedure described in Section 2.3 with step size of 1 and window lengths ranging from 4 to 16. We show the DICE coefficients of the inferred time courses in Figure C.1a. We see the optimal window length of six samples does not give perfect DICE coefficient. If we look closely and compare the simulated and inferred time courses in Figure C.1b, we observe that sliding window approach does a decent job at inferring relatively long state changes, but fails to infer the transient state changes accurately. This is the reason why we turn to generative models that implicitly learn the time scale from data.

We now train M‐DyNeMo and DyNeMo on the same simulated dataset. From Figure C.2a, we see that M‐DyNeMo can recover both state time courses accurately while DyNeMo cannot, due to its intrinsic model assumption of shared dynamics. Specifically, DyNeMo only infers the correlation dynamics. A quantitative measure of the accuracy of inferred time courses measure by DICE coefficient is shown in Figure C.2b, where we see M‐DyNeMo achieves perfect inference of both time courses. In this simulation study, the signal from time‐varying power is relatively low compared to that from time‐varying FC, which is why DyNeMo ignores the power dynamics. Additionally, we can investigate the reconstruction error measured by Riemannian distance between the reconstructed covariances and the ground truth at each time point, shown in Figure C.2c. It is clear that M‐DyNeMo has a lower reconstruction error than DyNeMo, indicating M‐DyNeMo is a more accurate model of time‐varying covariances on this data.

### Simulation 2: M‐DyNeMo Can Infer Accurately on Single Dynamics Data

It is important to show that the separate dynamics is not an artefact invented by M‐DyNeMo. This can be shown by training M‐DyNeMo on the second simulated dataset where data were simulated with a single underlying dynamics shared by both standard deviation and correlation. Shown in Figure C.3a, both of the M‐DyNeMo inferred mode time courses match the ground truth and the reconstruction error of time‐varying covariances is comparable to that of DyNeMo, shown in Figure C.3b.

## Appendix D Post Hoc Analysis

### Renormalising Mode Time Courses

As mentioned in (Gohil et al. 2022), the inferred mode time courses do not account for the difference in the relative magnitude of each of the spatial modes. For α, the raw mode time course is multiplied by the mean of the square of the diagonal elements of $E_{j}$ before normalised by dividing the sum over modes at each time point. Similarly for β, the raw mode time course is multiplied by the mean of absolute off‐diagonal elements of $R_{j}$ and normalised.

### Maximum Statistic Permutation Test for Spatial Map Similarity

The underlying assumption of this test is that sequences of mode time courses are exchangeable under the null hypothesis and we used the maximum statistic approach to correct for multiple comparison. The procedure for generating samples from the null distribution is as follows:

- Separate mode time courses into sequences of 1 s.
- Shuffle the sequences.
- Regress TV variances/correlations/covariances on the shuffled mode time courses and get regressed spatial maps.
- Match the spatial maps with cosine similarity using the Hungarian algorithm (Kuhn 1955).
- Get the maximum cosine similarity across pairs of modes.

The p‐value of the cosine similarities of each pair of modes is calculated as the percentage of samples from the null distribution that exceeds the observed cosine similarity. In this paper, tests with p‐value lower than 0.05, 0.01 and 0.001 are annotated with one, two, and three asterisks respectively.

## Appendix E More Results on Real Data

Here, we show some additional results on real MEG data in Figures E.1, E.2, E.3, E.4 and E.5.
